# The 5.5-year results of MegaOATS – autologous transfer of the posterior femoral condyle: a case-series study

**DOI:** 10.1186/ar2439

**Published:** 2008-06-16

**Authors:** Sepp Braun, Philipp Minzlaff, Regina Hollweck, Klaus Wörtler, Andreas B Imhoff

**Affiliations:** 1 Department of Sportsorthopaedics, Technical University Munich, Connollystraße 32, 80809 Munich, Germany; 2 Department for Medical Statistics and Epidemiology, Technical University Munich, Klinikum rechts der Isar, Ismaninger Straße 22, 81675 München, Germany; 3 Department of Radiology, Technical University Munich, Klinikum rechts der Isar, Ismaninger Straße 22, 81675 München, Germany

## Abstract

**Introduction:**

Large osteochondral defects of the weight-bearing zones of femoral condyles in young and active patients were treated by autologous transfer of the posterior femoral condyle (large osteochondral autogenous transplantation system (MegaOATS)). The technique presented is a sound and feasible salvage procedure to address large osteochondral defects in weight-bearing zones.

**Methods:**

Thirty-six patients between July 1996 and December 2000 were included. Thirty-three patients (10 females, 23 males) were evaluated by the Lysholm score and X-ray scans. A random sample of 16 individuals underwent magnetic resonance imaging analysis. The average age at the date of surgery was 34.3 (15 to 59) years, and the mean follow up was 66.4 (46 to 98) months. The mean defect size was 6.2 (2 to 10.5) cm^2^, in 27 patients affecting the medial femoral condyle and in six patients affecting the lateral femoral condyle. Trauma or osteochondrosis dissecans were pathogenetic in 82%.

**Results:**

The Lysholm score in all 33 individuals showed a highly significant increase from a preoperative median 49.0 points to a median 86.0 points (*P *≤ 0.001). Twenty-seven patients returned to recreational sports. X-ray scans showed a rounding of the osteotomy edge in 24 patients, interpreted as a partial remodelling of the posterior femoral condyle. Preoperative osteoarthritis in 17 individuals was related to significant lower Lysholm scores (*P *= 0.014), but progression in 17 patients did not significantly influence the score results (*P *= 0.143). All 16 magnetic resonance imaging examinations showed vital and congruent grafts.

**Conclusion:**

Patients significantly improve in the Lysholm score, in daily-life activity levels and in return to recreational sports. Thirty-one out of 33 patients were comfortable with the results and would undergo the procedure again. The MegaOATS technique is therefore recommended as a salvage procedure for young individuals with large osteochondral defects in the weight-bearing zone of the femoral condyle.

## Introduction

Large osteochondral lesions in young and active patients are a highly demanding challenge for orthopaedic surgery. There are commonly used procedures for the osteochondral transfer (for example, osteochondral autogenous transplantation system) from nonweight-bearing zones of the knee into the defect site with good results. These techniques, however, are limited by the defect size for harvesting reasons. In the case of osteochondrosis dissecans the lesions often exceed the size that can be treated by transfers of multiple osteochondral cylinders. As there are encouraging good results after osteochondral transplantations with single and multiple small cylinders in the weight-bearing zone of the femoral condyle up to an approximately 2 × 2 cm^2 ^defect size [1-4], there was a need for a technique that could be applied in the case of lesions larger than 4 cm^2 ^[5-7].

Autologous transfer of the posterior femoral condyle can provide autografts large enough to cover these defects, published for the first time in 1964 by Wagner [8] and later by Müller [9]. The transfer of the autologous posterior femoral condyle has been performed since 1996 at the senior author's institution as an alternative procedure to arthroplasty, and later was enhanced to the large osteochondral autogenous transplantation system (MegaOATS) technique, implementing the MegaOATS workstation in 1999. In the period July 1996 to March 2006, 102 individuals underwent this procedure.

In Europe, allografts are not rampant, are scarcely accepted by patients and are at least difficult and highly expensive to obtain. The purpose of the index procedure is therefore surgical treatment of larger osteochondral lesions with autografts. The results have been evaluated after a mean follow up of 5.5 years and are presented in the current paper.

## Materials and methods

All patients participating in the present study were educated in detail about the surgical technique and all alternative procedures with their advantages and disadvantages, and all participants chose to undergo the index surgical procedure. All participants signed informed consent to participate in follow-up examinations including radiographs and magnetic resonance tomography. The university hospital's institutional review board approved all aspects of the study.

All authors have read and agreed to the content of this manuscript and agree to free distribution to academic colleagues.

### Indications/contraindications

**Figure 1:**
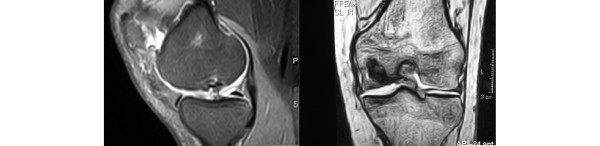
Preoperative magnetic resonance imaging scan of a deep osteochondral lesion. The left image shows the lesion in a sagittal view, eliciting the cartilage damage and the subchondral sclerosis. The right image shows shows the extension of the bone defect in a coronal image.

The indications for the index procedure in this series were Outerbridge grade IV osteochondral lesions [10], large osteochondrosis dissecans with nonvital or loose fragments (A/B International Cartilage Research Society osteochondrosis dissecans grade III and IV) [11], and focal osteonecrosis in the weight-bearing zone of the femoral condyle larger than approximately 4 cm^2 ^or osteochondral lesions that could not be addressed by standard osteochondral transfer techniques for other reasons (for example, depth) (Figure 1).

The main exclusion criteria were advanced osteoarthritis, significant narrowing of the joint lines and grade 2–4 osteoarthritic changes in more than the affected compartment. Deviation of the mechanical axis to the affected compartment was a criterion for performing a high tibial osteotomy (HTO). The alignment correction was planned and performed so that the mechanical axis was at 62% of the width of the tibial plateau, unloading the index femoral condyle.

### Surgical technique

The surgical technique originally combined the press-fit idea of osteochondral transfer plugs with the transfer of the posterior femoral condyle, which was performed freehand in the initial subgroup of the study and needed graft fixation with a minifragment screw [8]. The development of a special workstation allowed tailoring of a precisely cut transfer cylinder, which enabled secure press-fit fixation [12].

Surgery was performed under general anaesthesia with the patients in a supine position. A tourniquet was used to improve the intraoperative control of bleeding. Prepping and draping was performed in the usual sterile fashion.

The first steps of surgery were identical for both subgroups. A central incision and an anteromedial approach to expose the knee joint were performed.

**Figure 2:**
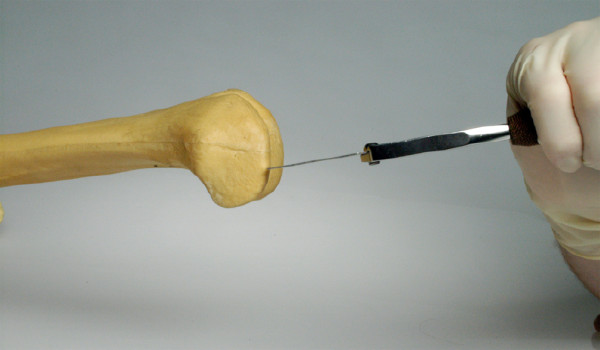
Harvesting the posterior femoral condyle with a chisel.

Before harvesting the posterior femoral condyle for transplantation, the defect was marked and its diameter was measured exactly. A k-wire was drilled in the centre of the lesion and then the graft's bed was prepared with a trephine over the k-wire. The trephine's diameter was available in 5 mm steps from 20 mm to 35 mm. Milling was performed as deep as healthy bleeding bone appeared. The depth was subsequently measured and the ipsilateral femoral condyle was harvested in about 130° of knee flexion with a chisel osteotomy according to the required graft depth. Two Hohmann retractors were placed medially and laterally to avoid injuries of the posterior joint capsule and of the cruciate and collateral ligaments (Figure 2). This procedure allowed harvesting of a graft that can be tailored to a cylinder up to 35 mm diameter and 20 mm thickness in adults.

In the first group, which underwent surgery in the time period from 1996 to 1999, the graft was sized freehand with a chisel to fit the priorly milled bed. This technique required graft fixation with a centered minifragment screw. After drilling the hole for the minifragment screw, a second drill of larger diameter was used as a countersink to put in the screw flush with the adjacent cartilage.

**Figure 3:**
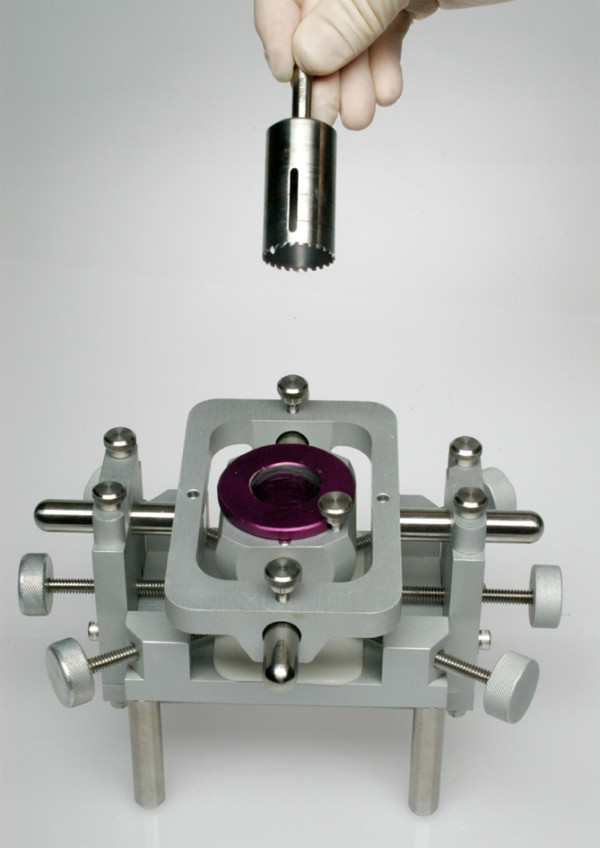
Workstation and hollow drill for sizing the graft.

For the subsequent subgroup, the graft was sized in a special MegaOATS workstation (Arthrex Inc., Naples, FL, USA) allowing the graft to be fixed with six positioning screws for precisely millcutting the cylinder (Figure 3). Consequently, the prior mentioned press-fit fixation without a screw was enabled.

**Figure 4:**
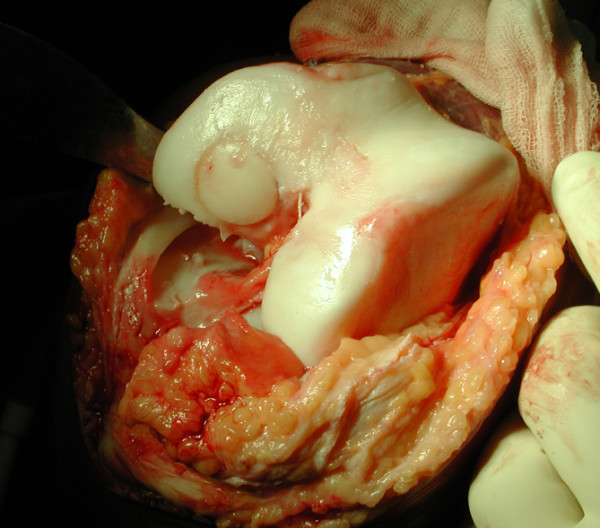
Large osteochondral autogenous transplantation system graft press-fit placed in the prepared defect site.

As the curvature of the posterior condyle in the sagittal plane is smaller than in the weight-bearing zone but is comparable with the coronal plane of the weight-bearing zone of the condyle, the graft in some cases was rotated 90° for a flush fit (Figure 4). In a few cases with an osteochondral defect far posterior close to the osteotomy, there was not sufficient bone support for press-fit fixation. A fixation of the graft with a minifragment screw in the previously described fashion was therefore necessary.

The technique required strict nonweight-bearing of the knee for 6 weeks. After this period, an arthroscopic screw removal was necessary. In those patients with a mechanical misalignment, a correction was performed with a closed-wedge HTO and an L-shaped plate in the same session.

The postoperative protocol was 6 weeks of nonweight-bearing on crutches and limited flexion up to 90°. Continuous passive motion on a motor splint for the time of nonweight-bearing was recommended for at least 4 hours/day. After this period an increasing load of 20 kg/week up to the patient's body weight and progressive range of motion followed. Full weight-bearing and a free range of motion were allowed 10 weeks postoperatively.

Beginning in the fourth month after surgery, patients were allowed to focus on specific training for their sport, especially improving proprioception and specific exercise patterns. Comeback to recreational sports was allowed and encouraged 6 to 9 months after surgery.

### Patients

From 1996 to 1999 the transfer of the posterior femoral condyle was performed freehand. The enhanced MegaOATS technique was introduced in July 1999.

Seventeen patients underwent the posterior femoral condyle transfer, one of them in both knees. In the following years (August 1999 until March 2006), 83 individuals were surgically treated in 84 cases (one patient in both knees) with the MegaOATS technique.

To evaluate the longest follow up possible, the first 36 cases operated between July 1996 and December 2000 were included in the study. Three individuals could not have been re-evaluated: one patient was in a coma vigil, another patient was untraceable and the third patient refused to join the re-evaluation. The retrieval rate for 33 out of 36 individuals was 91.7%.

The study collective includes 10 female and 23 male individuals, with six posterior condyle transfer (PCT) and four MegaOATS procedures in the females, and 11 PCT and 12 MegaOATS procedures in the males. The average age of all individuals (PCT and MegaOATS) included at the point of surgery was 34.3 years (range, 15 to 59 years; standard deviation (SD), 12.7), and the age was 39.8 years (range, 20 to 64 years; SD, 12.64) at the point of re-evaluation.

The mean follow up for the whole study group was 66.4 months (range, 46 to 98 months; SD, 13.2). It is obvious that individuals operated in the PCT technique have a longer follow up of 77 months (range, 62 to 98 months; SD, 9.3) versus 55.2 months (range, 46 to 62 months; SD, 4.9) for the MegaOATS technique.

The mean defect size for all individuals in the study group was 6.2 cm^2 ^(range, 2 to 10.5 cm^2^; SD, 1.8), located in 27 patients in the weight-bearing zone of the medial femoral condyle and in six patients in the lateral femoral condyle.

The average lesion size of PCT patients measured 6.8 cm^2 ^(range, 2 to 10.5 cm^2^; SD, 1.9). The patient with the relatively small but deep osteochondral lesion of 2 cm^2 ^in the medial femoral condyle had a congenital cartilage deficit in the femoral trochlea, which excluded him from being treated with an osteochondral plug transfer from that area into the defect site and autologous chondrocyte transplantation. The average defect size in the MegaOATS subgroup was 5.3 cm (range, 3.1 to 7.1 cm^2^; SD, 1.4).

The osteochondral lesions were of traumatic origin in nine patients. Osteochondrosis dissecans was pathogenetic for symptomatic lesions in 18 patients. The remainder of osteochondral lesions were due to defects after meniscal surgery (*n *= 2), due to aseptic necrosis of the subchondral bone (*n *= 2) or were idiopathic after multiple previous surgeries (*n *= 2).

Only nine individuals in the study group did not have previous knee surgery, whereas the remainder of the group had up to six surgeries before the PCT/MegaOATS procedure. Patients with PCT had an average of 1.9 (range, 1 to 6) versus 1.1 (range, 0 to 3) surgeries for the MegaOATS group before the index procedure. In 29 individuals, additional surgical interventions were necessary in the same session. A HTO was performed in 15 of 33 cases to unburden the medial compartment and to prevent the transplant from overload.

Eight lesions were deeper than the maximum depth of the MegaOATS cylinder and had to be supported by an additional cancellous bone graft from the head of the tibia. In seven cases it was additionally necessary to cover extra lesions by an osteochondral transfer from the lateral femoral trochlea besides the main cylinder. One individual had chondral lesions besides the main defects exceeding the maximum size of the MegaOATS in both knees, and thus was additionally treated by an autologous chondrocyte transplantation. Microfracturing of an additional cartilage lesion was performed in one individual.

Table 1 summarizes the patient data, prior surgeries and information about the procedures performed with the index surgery.

**Table 1:** Summary of patient data, surgeries prior to the index procedure and additional procedures

Patient	Age at surgery (years)	Follow up (months)	Lesion size (cm^2^)	Localization	Prior surgeries	Prior procedures^a^	Index procedure	Additional procedures
1	39	70	6	Medial Fc	2	B, C + I	PCT	Recorrection of tibial tuberosity, removal of bone spurs
2	21	78	10.5	Medial Fc	2	F, J	PCT	HTO 6°, cancellous bone grafting
3	28	72	7.1	Medial Fc	3	B, E, A + K	PCT	OATS lateral femoral condyle
4	38	62	8	Medial Fc	2	A, D	PCT	HTO 8°, OATS trochlea
5	37	67	7.1	Medial Fc	2	L, A	PCT	HTO 6°
6	20	72	7.5	Medial Fc	2	E, F	PCT	HTO 5°, cancellous bone grafting
7	21	77	2	Lateral Fc	0		PCT	Patella realignment distal and soft tissue
8	19	78	8.75	Medial Fc	2	A + B +C, D	PCT	HTO 6°
9	18	98	7.1	Medial Fc	0		PCT	HTO 6°, cancellous bone grafting
10	32	70	8	Medial Fc	6	2 × D, 3 × G, C	PCT	HTO 6°, partial meniscectomy, revision ACL reconstruction
11	34	67	9	Medial Fc	0		PCT	Cancellous bone grafting
12	35	82	7.1	Lateral Fc	5	A, B, E, C, D	PCT	
13	33	96	7.1	Medial Fc	2	A+B, E	PCT	OATS trochlea, ACI lateral femoral condyle
14	34	82	7.1	Medial Fc	1	A + B	PCT	ACI medial femoral condyle, partial synovectomy
15	56	81	6	Medial Fc	1	H + C	PCT	Cancellous bone grafting, arthrolysis
16	42	75	6	Medial Fc	3	G, C, D + A	PCT	Fulkerson procedure, medial and lateral release, OATS patella
17	26	82	3.75	Medial Fc	1	B + C + E	PCT	Cancellous bone grafting with bone cylinders
18	55	55	7.1	Medial Fc	0		MegaOATS	HTO 6°, partial meniscectomy
19	42	62	4.9	Lateral Fc	2	M + C, D	MegaOATS	
20	47	61	4.9	Medial Fc	0		MegaOATS	HTO 6°
21	17	49	4.9	Medial Fc	2	A + B, H	MegaOATS	
22	31	56	4.9	Medial Fc	2	A, B	MegaOATS	HTO 6°
23	34	59	4.9	Lateral Fc	0		MegaOATS	Cancellous bone grafting
24	59	58	3.1	Lateral Fc	0		MegaOATS	OATS patella, partial synovectomy
25	59	53	4.9	Lateral Fc	0		MegaOATS	OATS trochlea, partial synovectomy
26	44	49	4.9	Medial Fc	1	A + C + D	MegaOATS	HTO 8°, OATS trochlea
27	15	54	7.1	Medial Fc	1	E	MegaOATS	HTO 5°
28	23	46	3.1	Medial Fc	1	A + B	MegaOATS	
29	57	60	4.9	Medial Fc	1	D	MegaOATS	HTO 6°
30	28	61	7.1	Medial Fc	1	A + B + C	MegaOATS	HTO 6°
31	40	51	4.9	Medial Fc	0		MegaOATS	Partial synovectomy, lateral release
32	28	50	7.1	Medial Fc	2	E, B	MegaOATS	HTO 6°
33	20	59	7.1	Medial Fc	3	F, F, A	MegaOATS	Cancellous bone grafting

ACI, autologous chondrocyte implantation; ACL, anterior cruciate ligament; Fc, femoral condyle; HTO, high tibial osteotomy; MegaOATS, large osteochondral autogenous transplantation system; OATS, osteochondral autologous transplantation; PCT, posterior condyle transfer. ^a^A, arthroscopy; B, removal of loose bodies; C, cartilage smoothening; D, meniscal surgery; E, drilling of osteochondrosis dissecans; F, refixation of osteochondrosis dissecans; G, anterior cruciate ligament reconstruction; H, foreign body removal; I, distal patella realignment; H, bone biopsy; J, cancellous bone grafting; K, removal of bursa; L, Open reduction and internal fixation of fracture of distal femur with joint fracture; M, removal of a bone cyst.

All 33 patients were evaluated preoperatively and postoperatively standardized using the Lysholm score [13,14]; 29 were examined radiologically and clinically at the latest follow up. The first 16 out of 33 individuals who could be contacted by telephone and were scheduled for the re-evaluation examination were evaluated by standardized magnetic resonance imaging (MRI) scans, using the same 1.5 Tesla machine with identical settings for all sequences. The homogeneity of both groups – the PCT group and the MegaOATS group represented by eight individuals each – was reviewed by matching all data collected (Table 2).

**Table 2:** Comparison of random test groups undergoing magnetic resonance imaging (MRI) with patients not undergoing MRI

Parameter	Group	*n*	Mean value	Standard deviation	*P *value
Current age (years)	No MRI	17	43.94	14.37	
	MRI	16	35.50	9.59	
	Total	33			0.127
Follow-up (months)	No MRI	17	69.41	14.71	
	MRI	16	63.25	11.57	
	Total	33			0.260
Lesion size (cm^2^)	No MRI	17	6.43	1.43	
	MRI	16	5.91	2.21	
	Total	33			0.423
Number of prior surgeries	No MRI	17	1.59	1.68	
	MRI	16	1.44	1.00	
	Total	33			0.736
Lysholm score preoperatively	No MRI	17	49.65	17.58	
	MRI	16	49.38	18.62	
	Total	33			0.986
Lysholm score currently	No MRI	17	80.12	19.61	
	MRI	16	83.75	13.51	
	Total	33			0.763

### Statistical methods

Coherent data of ordinal scaled variables were tested using Spearman's correlation coefficient. Statistical significance was tested with the Wilcoxon test for related and nonrelated samples. The level of significance α was preset for all tests at *P *< 0.05.

As the MegaOATS procedure is a further development of the transfer of the posterior femoral condyle (PCT), but is not a vital change, both techniques are presented as one. Separate analysis of the two subgroups did not show significant differences, as will be shown later on.

## Results

Postoperative complications occurred in three individuals, but were of no negative consequence after treatment (muscle vein thrombosis, effusion after tumbling, inflammation of skin incision).

### Subjective satisfaction

Thirty-one out of 33 individuals (93.9%) questioned were subjectively highly satisfied with the results after surgery and assured that they would undergo the same procedure again if they were in the same situation as at that time. From their subjective point of view, patients stated overall improvement of knee function of an average 89% (range, 70% to 100%; SD, 10.7), on a scale with 0% being knee function not allowing one to participate in normal daily-life activities and 100% representing a knee function that allowed the patient all activities, including sports, without any limitations at the same level as before the injury. Two patients did not subjectively benefit from surgery and were subjectively not satisfied with their outcome.

### Score results

**Figure 5:**
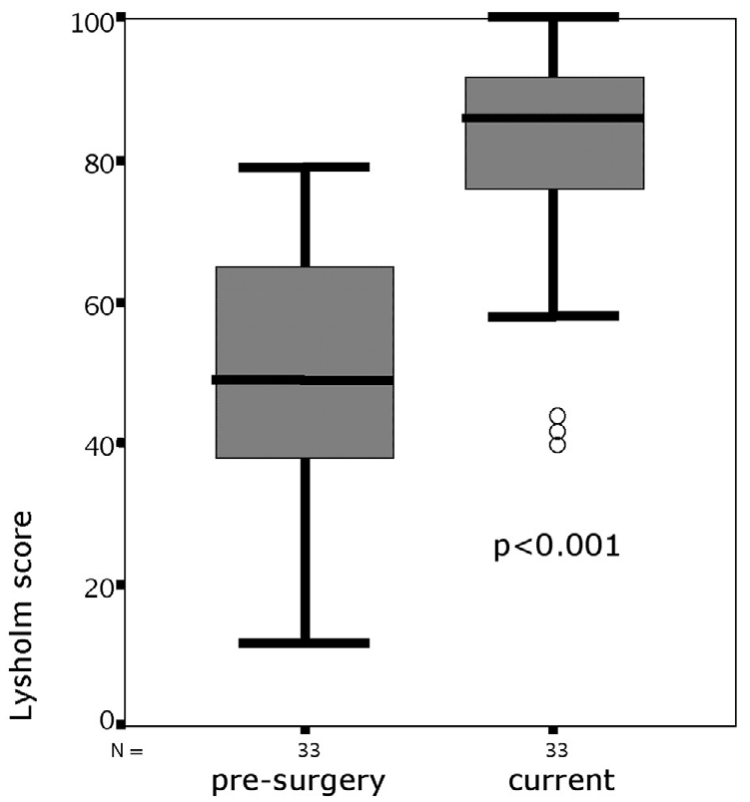
Preoperative and current Lysholm score after a mean 66.4 months. Box and whisker plot; circles, outliers.

The Lysholm score showed a highly significant increase in all but one individual of the study group; 32 out of 33 patients improved from a preoperatively median 49.0 points (range, 12 to 79 points; SD, 17.8) to a median 86.0 points (range, 40 to 100 points; SD, 16.8) (*P *< 0.001) after a mean 66.4 months (Figure 5 and Table 3).

**Table 3:** Lysholm score data preoperatively and at current examination

	*n*	Average	Standard deviation	Minimum	Maximum	Quantile		
						
						25%	50% (median)	75%
Lysholm score preoperatively	33	49.52	17.805	12	79	37.50	49.00	65.00
Lysholm score currently	33	81.88	16.772	40	100	73.50	86.00	93.00

The minimal individual increase in Lysholm score was 4 points, and the maximum was 78 points. One patient solely did not improve his score. This patient was evaluated retrospectively for the time before implantation of a total knee arthroplasty at the age of 62 because of constant pain 5 years after the PCT.

**Figure 6:**
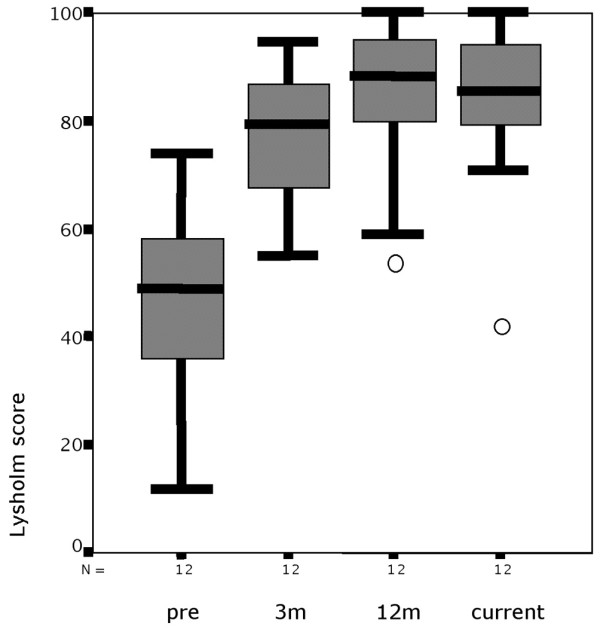
Development of the Lysholm score. The Lysholm score at presurgery, at 3 months (3 m) and 12 months (12 m) postoperatively, and at the current examination. Box and whisker plot; circles, outliers.

Twelve patients out of the 33 individuals included showed up for both recheck examinations at 3 and 12 months after surgery. For these 12 patients a consistent dataset preoperatively, at 3 months and at 12 months can be compared with a current score at an average of 74 months (range, 58 to 98 months; SD, 13.5). There is a marginal decrease in median scores for this group from a median 88.5 points after 12 months to a current score of 85.5 points, but the group shows significant improvement at every stage compared with the preoperative score (*P *= 0.006 at 3 months, *P *= 0.003 at last examination) (Figure 6).

A HTO was performed in 15 individuals with pre-existing malalignment. The score outcome with and without correction of axis by a HTO showed no significant difference (Table 4).

**Table 4:** Lysholm score results of patients with and without high tibial osteotomy

Month	No high tibial osteotomy			High tibial osteotomy		
	
	*n*	Median	Standard deviation	*n*	Median	Standard deviation
1	18	51	16	15	48	20
4	11	75	12	7	79	12
7	5	67	21	6	75	25
13	9	87	13	7	87	15
19	4	92	3	2	99	1
67	18	82	14	15	82	20

### Daily and sports activities

All individuals evaluated were of normal or high activity levels before the knee lesions became symptomatic. All but one individual reported sports activities from at least once a week to daily workouts on recreational to semiprofessional levels before the knee damage became symptomatic. Immediately before surgery, 29 out of 33 participants were not able to perform any sports and were massively limited in their daily live activities.

**Figure 7:**
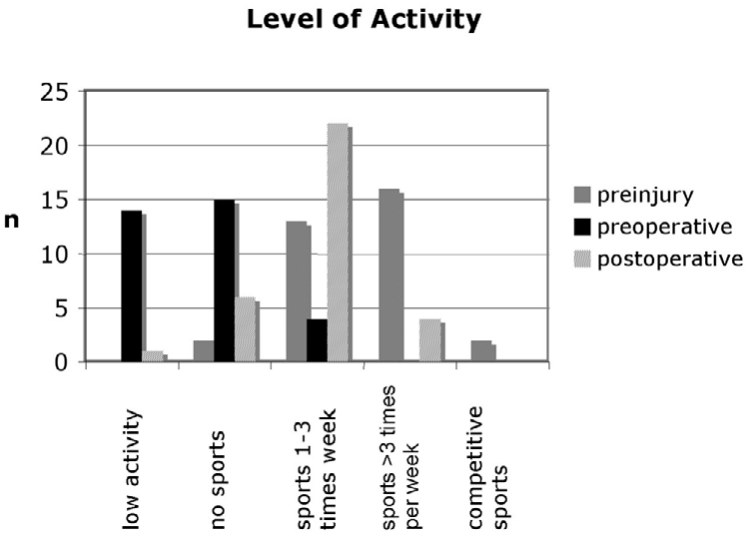
Level of activity of the patients. Patient activity levels prior to the knee injury, at presurgery and at the current re-evaluation.

Twenty-seven of the patients returned to sports activities on a recreational level regularly (Figure 7), such as road cycling, Nordic walking, cross-country and alpine skiing and swimming. One individual returned to playing soccer in a higher league.

### Physical examination findings

Three individuals showed positive clinical signs of medial meniscus degeneration. Proving the clinical suspicion by MRI, two of the participants had a meniscal tear.

There was no clinical evidence for medial or lateral collateral instability in all knees tested. Twenty-three of the individuals tested presented anteriorly, posteriorly and collateral stable knees at the current examination. Five patients had a positive Lachman test, but presented with a firm endpoint. One patient showed a positive Lachman test without a firm endpoint and without a positive pivot shift test.

In three individuals there was the first diagnosis of anterior instability at the point of the current examination, without adequate trauma after the MegaOATS/PCT procedure. In two cases an anterior cruciate ligament lesion 10 years and 2 years prior to the procedure, respectively, led to instability. One patient tore the anterior cruciate ligament playing beach volleyball 3.5 years after surgery.

The range of motion was documented in comparison with the noninvolved knee. Twenty-four patients presented with full extension. Compared with the nonsurgical side, one patient had a bilateral extension deficit of 15° before surgery and was measured with a -10° extension after the procedure. An extension deficit of 5° was evaluated in four subjects, two of whom were documented prior to surgery. Both 10° and 15° extension deficits were found in two patients each, and for both the 10° and 15° deficits this extension was documented prior to the procedure in one individual.

The flexion of the surgical knees was full compared with the uninjured knee in 16 individuals. Six individuals had a 5° flexion deficit, which was already noted prior to the procedure in four of these patients. A flexion deficit of 10° was documented in eight participants and a deficit of 20° in three subjects, of which three cases and two cases, respectively, were pre-existing before surgery.

Twelve patients had no difficulties and 14 patients had minor difficulties with deep squatting. Seven individuals could not squat with more than 90° knee flexion at the current physical examination. Sixteen patients had no trouble climbing stairs, and another 16 participants reported minor difficulties. Only one patient reported that he had significant difficulties climbing stairs.

### Radiographic results

The state of osteoarthritis was evaluated after Jäger and Wirth [15], a radiographic grading system accepted and commonly used in Europe, staging osteoarthritis from grade I to grade IV (Table 5).

**Table 5:** Jäger and Wirth classification for osteoarthritis of the knee [15]

Grade	
1	Initial osteoarthritis with hinted osteophytes at the eminentia intercondylaris and the articular side of the inferior and superior pole of the patella
2	Moderate osteoarthritis with hinted osteophytes at the tibia plateau, moderate narrowing of the joint space, hinted flattening of the femoral condyles, moderate subchondral sclerosis
3	Advanced osteoarthritis with 50% narrowing of the joint space, manifest flattening of the femoral condyles, osteophytes at the tibial plateau, tibial spine, intercondylar notch and at the articular side of the inferior and superior pole of the patella. Significant subchondral sclerosis
4	Pronounced osteoarthrosis. Joint destruction with significant narrowing of the joint space or loss of joint space, disturbed contour of the bone margins. Cystic changes in the tibia plateau, femoral condyles and patella. Subluxation of the tibia to the femur

There was a positive correlation between patient age and grade of osteoarthritis before surgery (*P *< 0.001) and at the point of follow-up examination (*P *< 0.001).

**Figure 8:**
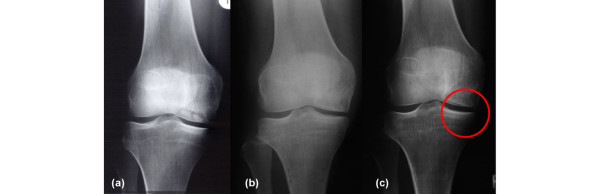
Progression of osteoarthritis in X-ray examinations. Progression of osteoarthritis in Patient 6: (a) 5 months before surgery, (b) 12 months postoperatively and (c) 72 months postoperatively. The circle marks the medial compartment without osteoarthritic changes.

Twelve individuals of the collective showed no radiographic signs of osteoarthritis preoperatively, and eight of them also showed no signs in the current follow-up radiographs. Progression of osteoarthritis was seen in 17 patients, with pre-existing arthritis in 13 patients. Fifteen out of 17 individuals deteriorated by one grade (Figure 8). Twelve individuals had no progression of osteoarthritis and four showed initial signs of osteoarthritis at the point of current evaluation without pre-existing positive radiographic findings.

**Figure 9:**
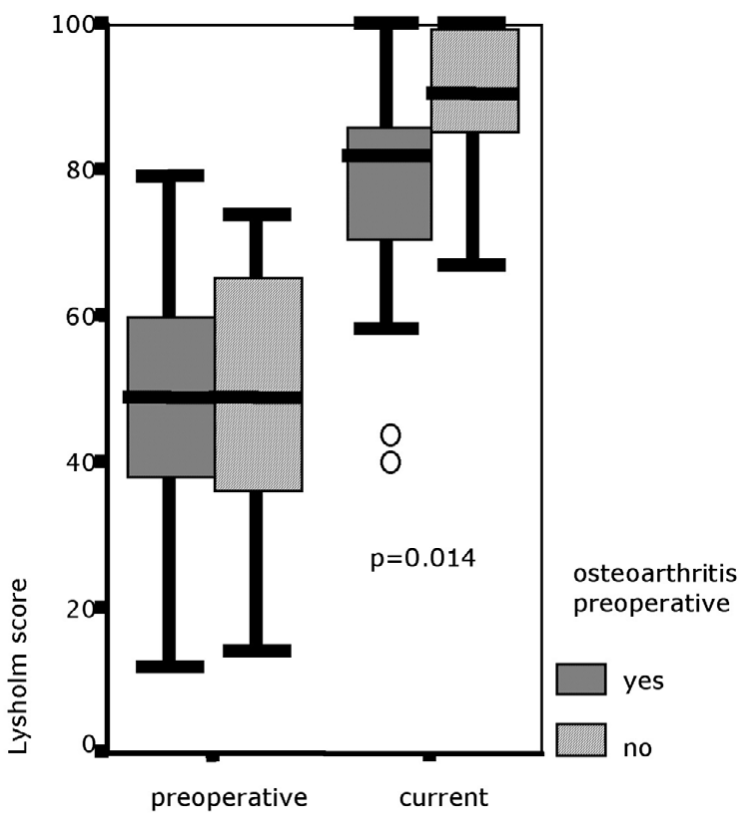
Lysholm score for patients with and without osteoarthritis documented presurgery. Box and whisker plot; circles, outliers.

Preoperative osteoarthritis was related to significant lower Lysholm scores (*P *= 0.014), but progression of pre-existing osteoarthritis did not significantly influence Lysholm score results (*P *= 0.143) (Figure 9).

**Figure 10:**
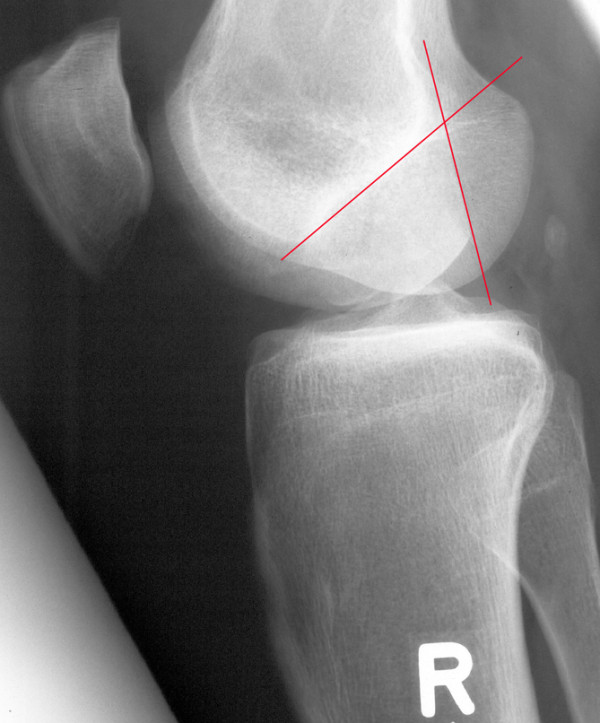
Sharp edge from the osteotomy for harvesting the graft, one red line marking the osteotomy of the posterior femoral condyle, the crossing line marking the Blumensaat's line: Patient 17, 2 months postoperatively. "R" marks that this is a right knee.

**Figure 11:**
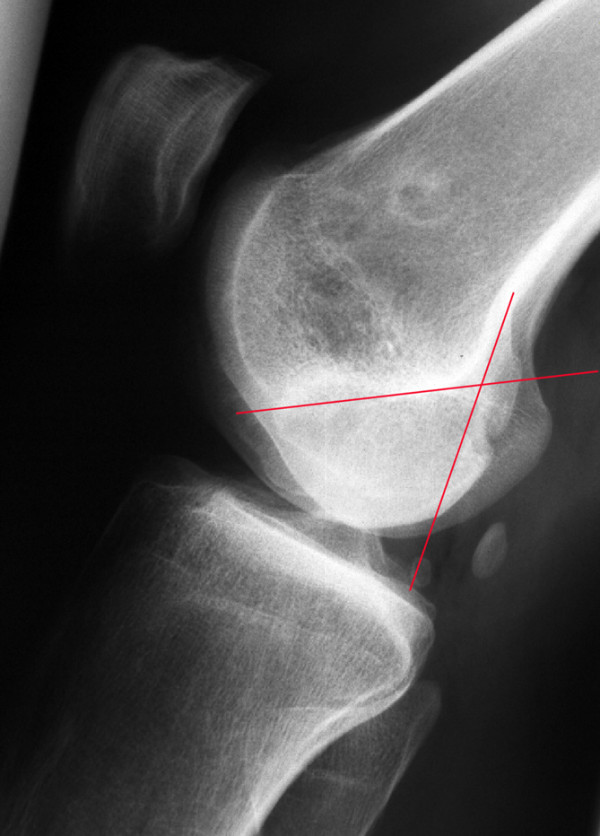
Partial remodelling of the posterior femoral condyle: Patient 17, 82 months postoperatively. One red line marks the prior osteotomy of the posterior femoral condyle according to figure 10, the crossing line marks the Blumensaat's line.

Postoperative radiographs showed a sharp edge from harvesting the posterior condyle (Figure 10) in all patients. X-ray examinations at the point of current evaluation showed a rounding of the osteotomy edge in 24 cases, interpreted as a partial remodelling of the posterior femoral condyle (Figure 11). This was also seen in MRI analysis.

### Magnetic resonance imaging findings

**Figure 12:**
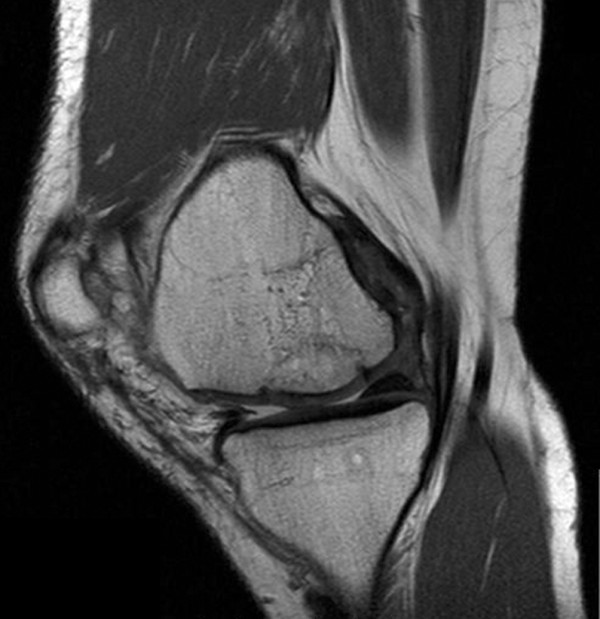
Magnetic resonance image after posterior condyle transfer. Magnetic resonance imaging scan of Patient 7, 72 months after posterior condyle transfer of the lateral femoral condyle.

**Figure 13:**
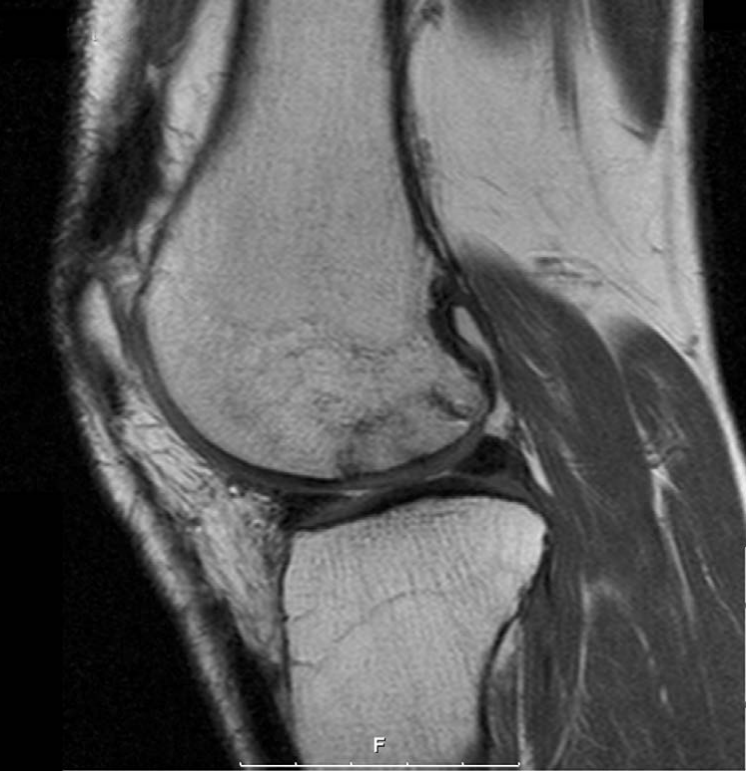
Magnetic resonance image after the large osteochondral autogenous transplantation system technique. Magnetic resonance imaging scan of Patient 23, 59 months after the large osteochondral autogenous transplantation system and cancellous bone grafting.

All MRI scans at the point of follow-up examination showed vital and congruent grafts. Thirteen patients had a signal identical to surrounding cartilage. Figures 12 and 13 show a representative current MRI for the PCT and MegaOATS procedures, respectively.

Three individuals had signal alterations in the cartilage surface, which could be estimated as Outerbridge grade I and grade II cartilage damage [10]. Subchondral bone signals were without pathological findings in 10 individuals, four participants showed bone edema and small bone cysts, and two individuals solely had small bone cysts.

The remainder of the knees examined by MRI showed no pathological findings in seven patients, grade I/II cartilage lesions in four patients, grade III lesions in three patients and grade IV lesions in two individuals in the compartments not treated by the index procedure. The subchondral bone was healthy in 14 patients, but two participants had small bone cysts – one ventral of the graft and the other at the medial tibial plateau.

There was no pathological signal for menisci in 12 individuals. Two patients had a partial resection of the medial meniscus. One of these patients had pre-existing degenerative signs and was operated on 3 years after the PCT; the patient is currently showing a re-rupture in the resected meniscus at the point of re-evaluation. One individual had degenerative signs. Another patient developed a new meniscal tear without pre-existing degenerative signs.

Fourteen out of 16 individuals who underwent MRI showed a partial remodelling of the posterior femoral condyle, which was seen in the range from rounding of the osteotomy edge with bone dense tissue to significant filling in of the harvesting defect.

### Matching posterior condyle transfer and the MegaOATS

Results of the PCT and the optimized technique of MegaOATS are presented together. The newer technique of MegaOATS is regarded as an improved surgical technique, making surgery easier and faster, but it does not change the basic principles of the procedure.

The individual patient data and medical histories showed no significant difference for PCT and MegaOATS, but the lesion size of 6.8 cm^2 ^for PCT was significantly larger compared with the 5.3 cm^2 ^for the MegaOATS (*P *= 0.005). There was a nonsignificant difference found for the number of prior surgeries, with 1.9 (range, 0 to 6; SD, 1.6) for the PCT group versus 1.1 (range, 0 to 3; SD, 1.3) for the MegaOATS group (*P *= 0.05).

A critical review of the data suggests that the lesions size could have been overestimated in PCT patients by surgeons. With introduction of the MegaOATS technique, the lesion size is measured by the diameter of the hollow trephine and therefore is much more precise.

Comparing scores for individuals treated with PCT and the MegaOATS, there was a significant difference (*P *= 0.001) in preoperative Lysholm scores (40.4 points for PCT to 59.3 for MegaOATS) but not for the follow-up evaluation (82.7 points for PCT to 81.0 points for MegaOATS, *P *= 0.828) (Table 6).

**Table 6:** Comparison of Lysholm score of the posterior condyle transfer (PCT) group and the large osteochondral autogenous transplantation system (MegaOATS) group

	Group	*n*	Mean value	Standard deviation	Minimum	Maximum	Quantile			*P *value
								
							25%	50% (median)	75%	
Lysholm score preoperatively	PCT	17	40.35	13.7	12	58	36	41	53.5	
	MegaOATS	16	59.25	16.7	19	79	49	65	70.7	
	Total	33	49.52	17.8	12	79	37.5	49	65	0.001
Lysholm score currently	PCT	17	82.71	16.6	40	100	73.5	85	96.5	
	MegaOATS	16	81.00	17,4	42	100	73.2	86	90	
	Total	33	81.88	16.8	40	100	73.5	86	93	0.828

Both the PCT and the MegaOATS techniques present similar score results at the final follow-up examination, but with a lower preoperative level for PCT. The improvement of PCT individuals was a mean 42.4 points (range, 13 to 78 points; SD, 18.3) after an average of 77 months (range, 62 to 98 months; SD, 9.3). The score improvement for the MegaOATS group after an average of 55.2 months (range, 46 to 62 months; SD, 4.9) was 21.7 points (range, -7 to 68 points; SD, 17.0), which is a significant difference in score improvement (*P *= 0.002).

## Discussion

The MegaOATS technique can be indicated in cases that require treatment of large femoral osteochondral lesions in the weight-bearing zone. Such large defects are of biomechanic relevance preoperatively. MegaOATS, as a salvage procedure, aims at painfree mobility of young patients, not at re-establishing a completely healthy joint.

We furthermore acknowledge that the presented patient population is heterogeneous, which reflects the situation of patients with an indication for a salvage procedure. It is common that a patient population with this type of complex knee injury or pathology presents with more than one isolated underlying pathology and thus needs more than one singular surgical procedure to address all of them. Nonetheless, even a surgical procedure like the presented PCT/MegaOATS technique can be indicated as the first surgical intervention in particularly severe cases.

In the context of a medical thesis, a previous study tested seven fresh-frozen cadaver knees biomechanically [16]. Five cadaver knees made up the normal group, and two knees made up the varus group with a varus deformity of 6° in both cases. In these two specimens, HTO was performed for correction of the malalignment. The experimental setup simulated a one-legged stand with the axial load of the donor's body weight. Using Fuji Prescale films, the intraarticular pressure and contact area was measured in 0°, 30°, 60° and 90° of flexion before and after harvesting the posterior femoral condyle. For the varus group, data were recorded before HTO, after HTO and after HTO combined with transfer of the posterior femoral condyle. Beginning with 30° of flexion there is an articulation of the edge deriving from harvesting the posterior condyle, and as a consequence thereof the contact area in the femorotibial joint is reduced. The minimum contact area is reached at 60° of flexion. The reduced contact area after transferring the posterior femoral condyle led to an increased intraarticular pressure, beginning at 30° of flexion and with a maximum at 60° of flexion. The biomechanical test showed the significant relevance of HTO in case of varus misalignment to unburden the affected joint compartment [16].

There is a widely accepted consensus that symptomatic cartilage damage usually needs surgical treatment to avoid early deterioration [2,15,17,18]. Traumatic cartilage defects or lesions of osteochondrosis dissecans in young and active individuals especially challenge orthopaedic surgeons. Therapy must make normal daily-life activities possible, and in ideal cases restore the individual's ability to at least perform sports on a recreational level.

Several surgical techniques for treatment of cartilage defects are described, but today only the transplantation of osteochondral cylinders can provide real hyaline cartilage on the one hand, and can replace necrotic subchondral bone on the other [19]. Mosaicplasty and osteochondral transfer are commonly known and applied techniques [1,20], but their adoption is limited by defect size [5,21].

There are good results published for five patients with 2.5 years of follow up, whose femoral osteochondral lesions in weight-bearing zones were covered by fresh-frozen femoral allografts in the MegaOATS press-fit technique [13]. McCulloch and colleagues recently reported good results after transplantation of prolonged fresh allograft osteochondral plugs [22], which have been stored at 4°C and preserve chondrocyte viability and potentially a reduced immune response [23,24]. These results are encouraging, but are not transferable to every patient population. At the present time, allografts are very difficult to obtain, are rarely accepted by patients and are, not least, extremely expensive in Europe. Patients fear transmission of viral infections or an immunological response (for example, tissue rejection) [25]. Moreover, current regulations of the European Union (EC directive 2004/23/EG) – which were recently implemented as national law in Germany, for example – put tissue allografts, such as bone and cartilage, under the control of the strict law of drugs. As a result, the availability osteochondral allografts worsened and surgeons have large-scale liability for the allograft.

Debriding the defect, stimulating the bone marrow by drilling, abrasion or microfracturing, cannot substantially reduce pain for the long term and does not address any bone defects [25,26]. Furthermore, bone marrow stimulation (for example, the microfracture technique) is not indicated in case of a subchondral bone defect or a loss of bone (osteochondrosis dissecans fragment) [27].

The Outerbridge technique, using the lateral patellar facet as an autograft, can cause femoropatellar pain and affects patella tracking and stability [28]. Autologous chondrocyte transplantation techniques require additional support of cancellous bone in order to replace necrotic or otherwise damaged subchondral bone [29]. Restoration of a congruent joint surface is therefore very difficult to achieve in surgery and might collapse with weight-bearing. Implementation of MegaOATS as further development of the transfer of osteochondral cylinders therefore enables treatment of far larger lesions up to 35 mm in diameter, respectively 9.6 cm^2^, by harvesting the posterior femoral condyle [6].

Showing good clinical results, patients significantly improve in their daily-life activity levels and return to recreational sports. The median Lysholm score improved by 37 points after 5.5 years and showed a highly significant increase at all stages of re-evaluation. Thirty-one out of 33 patients were comfortable with the results and would undergo the procedure again.

A difference was found in score development after the PCT and MegaOATS procedures. Starting from a lower level for PCT, individuals came to about the same score results at the current examination as those after MegaOATS. Because of encouraging good results seen in the first years of PCT follow up and because of considerable improvements of the surgical technique making the procedure considerably easier to handle, more individuals were surgically treated. Furthermore, the PCT patients had in average 0.8 more prior surgeries to the index procedure than the patients undergoing MegaOATS. This may in addition explain the lower Lysholm score at the starting point for the PCT group.

Autologous osteochondral transfer techniques are all associated with more or less large harvesting defects. Beyond doubt, MegaOATS comes with a large harvesting area, but remodelling of the harvested posterior femoral condyle was seen in most individuals. The previously mentioned biomechanical tests showed that loss of the posterior femoral condyle is of relevance from 30° to 60° of flexion, mainly influenced by the effects of the sharp harvesting osteotomy edge [6,16]. As the partial remodelling of the posterior condyle or rounding of this edge might reduce this influence, the sacrifice of the posterior condyle becomes less important.

Rounding of the osteotomy edge was seen in 24 (83%) individuals currently evaluated after a mean 66.4 months. This particular finding of rounding was seen for the first time at follow-up examinations 6 months postoperatively.

There still remains, however, room for discussion of whether the three meniscal lesions that appeared after the transfer of the posterior femoral condyle are in direct association with this intervention. All three individuals with positive clinical meniscal signs show a rounding of the osteotomy edge and partial remodelling of the condyle, so the lesions might be no direct result of the loss of the posterior condyle. Furthermore the lesions appeared 3 years, 4.75 years and 5.5 years after surgery in individuals with an above-average level of sporting activity, such as soccer, tennis and skiing, and therefore with a higher risk of injury than other individuals in the study group.

Preoperatively 12 (41%) individuals of the study group showed no radiographic signs of osteoarthritis. By the time of the last follow-up examination, eight patients (28%) were without evidence of osteoarthritis in X-ray examinations and 17 (59%) of all individuals showed progression, only four for the first time. Thirteen (76%) of those participants with progression had osteoarthritis before surgery. The deterioration of osteoarthritis was by one grade in all but two patients with progression. The prevalence of the first signs of osteoarthritis is described in about 50% of the European population aged between 30 and 50 years [15], which relativises this radiologic result.

As the Lysholm score results of the present study show, the outcome is limited by pre-existing osteoarthritis. General osteoarthritis must therefore be considered a contraindication to performing a transfer of the posterior condyle.

The MRI analysis in a random group of 16 patients showed vital and congruent transplants in all individuals – at least small bone cysts in marginal parts of transplants or surrounding bone appeared, presumably coming from microscopic metal wear of tools. Another hypothesis interprets these small cysts as partially necrotic tissue [30]. Histologic studies evaluating cartilage after osteochondral transplantation showed that there is no healing or ingrowth to the adjacent cartilage [31], but there is osseous integration of the graft [32]. In comparison with multiple small osteochondral plugs, the larger the graft is in diameter, the less the zone of nonunion of transplanted cartilage to adjacent cartilage. This smaller nonunion might reduce the histological observed cartilage degeneration [31] in osteochondral grafts, presumably because of reduced sheer forces.

Our results presented after 5.5 years have to be followed up, as the presented period is short compared with the life expectancy of young patients. We shall focus especially on degenerative changes, osteoarthritis and the status of the donor site. Filling-in of the harvested posterior femoral condyle was seen in the majority of the patients and will be subject to further studies.

Strategies for future revision cases or further surgical treatment potentially following the presented procedure will be focused on joint arthroplasty. As the posterior femoral condyle is an important structure for implanting some types of protheses, affected joints can be replaced either by models designed for ventral alignment or by revision systems with intramedullar fixation.

The main contraindication was osteoarthritis grade II to grade IV (Jäger and Wirth classification [15] (Table 5) in more than the compartment to be treated by the procedure. Osteochondrosis dissecans lesions exceeding a size of 35 mm in diameter and/or extending into the posterior condyle that will be used as a graft were not addressed by the MegaOATS procedure. Nevertheless, additional cartilage lesions or noncircular defects were treated with an additional osteochondral plug harvested from the femoral trochlea in the usual fashion in some cases.

Depending on the biological age, general bone quality and osteoarthritis of the patient, the MegaOATS procedure can be performed up to an age of about 55 years. As long as harvesting and implantation of the graft leaves the epiphysis intact, the technique can also be performed in young patients. In cases of misalignment correction of the axis (for example, HTO in the case of varus deformity), unloading the affected compartment is recommended. Patients with additional functional joint instability also need to be stabilized; for example, with an anterior cruciate ligament reconstruction.

## Conclusion

The MegaOATS technique is recommended as a salvage procedure for young individuals with large osteochondral defects in the weight-bearing zone of the femoral condyle who are drastically limited in performing normal daily activities – not to mention performing sports activities. In these individuals the MegaOATS procedure can restore pain-free daily mobility, and in many cases patients can come back to sports at a recreational level.

## Abbreviations

HTO = high tibial osteotomy; MegaOATS = large osteochondral autogenous transplantation system; MRI = magnetic resonance imaging; OATS = osteochondral autogenous transplantation system; PCT = posterior condyle transfer; SD = standard deviation.

## Competing interests

SB is currently working as a research fellow at a nonprofit research foundation in the USA. Arthrex Inc. (Naples, FL, USA) is financially supporting this research position. There was no financial support or funding for this study. The remaining authors declare that they have no competing interests.

## Authors' contributions

SB was responsible for the study design, performed physical examinations of the patients, reviewed the diagnostic tests and drafted the manuscript. PM was responsible for the follow-up examinations and the patient scheduling, worked on the statistical analysis and helped draft the manuscript. RH was responsible for the statistical analyses. KW was responsible for the analysis and assessment of all radiographic and MRI tests. ABI was responsible for the entire study, performing the majority of the surgeries and developing the surgical technique. All authors read and approved the final manuscript.
